# Factors associated with risky sexual behaviour among clients undertaking HIV testing and counselling services at a secondary referral hospital Lagos, Nigeria

**DOI:** 10.4314/ahs.v22i1.7

**Published:** 2022-03

**Authors:** Olusola Adedeji Adejumo, Bisola Ibironke Adebayo, Sunday Adesola, Abimbola Bowale, Esther Ngozi Adejumo, Stella Atewe, Olayinka Sijuade, Andrew Airauhi, Oluwajimi Sodipo, Yeside Shogbamimu

**Affiliations:** 1 Department of Community Health and Primary Health Care Lagos State University Teaching Hospital Ikeja Lagos Nigeria; 2 Mainland Hospital Yaba Lagos Nigeria; 3 Department of Medical Laboratory Science. Babcock University Ilishan Remo Ogun State Nigeria; 4 Department of Family Medicine. Lagos State University Teaching Hospital Ikeja Lagos Nigeria; 5 Department of Disease Control, Lagos State Ministry of Health Nigeria

**Keywords:** Risky sexual behaviour, associated factors, HIV counselling and testing

## Abstract

**Background:**

This study determined the prevalence of risky sexual behaviour and its associated factors among clients who accessed HIV counselling and testing services at a secondary referral hospital in Lagos, Nigeria.

**Methods:**

A retrospective review of clients' records was conducted. The Client Intake Form of people who accessed HIV counselling and testing services at Mainland Hospital in Lagos, Nigeria between July 1, 2016, and December 31, 2017, were reviewed. Multivariate analysis was conducted to determine the associated factors of risky sexual behaviour.

**Results:**

A total of 4273 client's records were analyzed, 3884 (90.9%) reported having sex before HIV counselling and testing (HCT). The prevalence of risky sexual behaviour among clients was 41.5%. More males and HIV positive clients had unprotected sex with a casual partner three months before HIV counselling and testing (p < 0.05). More singles than the married had unprotected sex with casual partners (p <0.001) and multiple sexual partners (p =0.002). The prevalence of risky sexual behaviour reduced with advancing age. Being single and having an HIV infection were associated with risky sexual behaviour in this study.

**Conclusion:**

Age, marital status and HIV status were associated factors of risky sexual behaviour.

## Introduction

Risky sexual behaviour (RSB) comprises various kinds of behaviours related to sexuality such as multiple sexual partners, premarital sex and unprotected sex which increases the likelihood of an individual to contract, HIV, STIs, unwanted pregnancy and unsafe abortions.^1^ This definition is not sacrosanct as authors have defined RSB according to the variables of interest. Other factors such as the age of sex debut, paid sexual partners, substance use, and risk of familiar conflicts or conflicts with the law have been considered by different authors in defining RSB.^2^

The World Health Organization (WHO) reported that 95% of the 14,000 people newly infected HIV daily are from developing countries.^3^ The prevalence of HIV varies across the globe depending on the settings. In high HIV prevalent settings like eastern and southern Africa, young women between 15 – 24 years accounted for 26% of new HIV infections in 2016. However, key populations (sex workers, transgender people, prisoners and men who sleep with men) and their partners constituted 80% of new HIV infection. 4

Nigeria has the second-largest HIV epidemic globally. In 2016, a Joint United Nations Program on HIV/AIDS (UNAIDS) report estimated the prevalence of adult HIV in Nigeria at 2.9%, 3.2 million people were reported to be living with HIV, 220,000 new HIV infections, 160,000 HIV related deaths and 1.8 million HIV orphan children were reported.^4^ Close to 80% of new HIV infections in Nigeria are due to heterosexual sex.^5^ Behaioural factors such as transactional sex, early sexual debut, extramarital sex, fatalism, syndrome of denial, poor condom use and alcohol have been pinpointed to drive HIV infection in Nigeria and other countries in sub-Saharan Africa.^6^ A national survey reported a high proportion of people engaged in risky sex such as multiple partnering, non-marital sex and un-protective sex in the country.^7^

HIV Counselling and Testing (HCT) has been identified as an HIV prevention strategy globally. It provides the opportunity for clients to know their HIV status, deals with HIV-associated anxiety, and motivates them to change (RSB) that will prevent HIV transmission. 8,9 The findings from studies on the association between HCT and sexual behaviours are not consistent, a study from Zimbabwe reported that RSB increased with HCT,^10^ but studies from Tanzania and Kenya observed a reduction in RSB after the uptake of HCT.^11^,^12^ A systematic review and meta-analysis from sub-Saharan Africa and the United States respectively opined that HIV infection was not substantially prevented by HCT when offered to HIV uninfected persons without their sexual partners but there was a strong and consistent condom uptake among HIV positive discordant couples.^13^,^14^ Amidst all the debates, while it is suggestivehat HCT could serve as a tool for promoting messages that can reduce RSB and ultimately HIV prevalence in high burden countries like Nigeria, 15 there is, therefore, a need to assess the RSB of clients accessing HCT in the country.

In Nigeria, RSB has been well researched especially among the adolescent and youths 16–19, to the authors' knowledge limited studies were conducted among clients assessing HCT services. 20,21 These studies focused on condom use and sexual characteristics of clients accessing HCT. There is a paucity of information on the prevalence and associated factors of RSB among clients accessing HCT in Nigeria. This present study which assessed the prevalence of RSB and associated factors among clients undertaking HCT services at a secondary referral hospital in Lagos, Nigeria is an attempt to fill the study gap.

## Methods

### Study design

A review of records of clients (Client Intake Form) that accessed HCT services at Mainland Hospital Lagos Nigeria between July 1, 2016, and December 31, 2017.

### Study background HIV counselling and testing in Lagos Nigeria

Mainland hospital is a secondary referral infectious disease hospital in Lagos Nigeria for the treatment of tuberculosis (TB), HIV, multi-drug resistant TB and other epidemic-prone diseases like cholera, Ebola virus diseases and Lassa fever. The hospital was established in 1930, however, treatment of HIV commenced in the hospital in 2000 with support from Implementing Partners. Over 12,000 clients had been enrolled for ARVs in the hospital as of the end of 2017.

HIV testing was routinely offered to every client presenting to the hospital in compliance with the National guidelines for HCT.^21^ HIV testing was usually preceded by counselling which in part assesses the HIV risk behaviours of clients. Post-test counselling was also offered to clients regardless of the HIV results. HIV negative clients were counselled on the window period for HIV infection, methods of HIV prevention, risk reduction, use of a condom, the importance of knowing the HIV status of sexual partners and the need for repeat testing every 6 – 12 months especially of high-risk clients. Test results were interpreted to HIV positive clients and assistance was offered to mitigate the emotions arising from the diagnosis. Information on anti-retroviral therapy (ART), its benefits, where and how to access ART was given. Adherence counselling, positive living, disclosure of status to the spouse, and prevention of HIV transmission were also provided. Where applicable, clients were encouraged to bring their spouses and children for HIV testing and provision was made for other support services such as screening and treatment for TB and STIs, prophylaxis for opportunistic infections, contraception and antenatal care as the case may be.^21^

Determine (determine HIV-1/2 Alere Determine™, Japan 2012) and Uni-GoldTM (Trinity Biotech PLC, Wicklow, Ireland 2013) were used for HIV diagnosis in series. Determine was used for HIV diagnosis while Uni-Gold was used to confirm positive results. A concordant result was regarded as positive, but STAT-PAK® was used as a tie-breaker in discordant cases.

### Assessment Tool

The tool used for the study was the National Client intake form designed nationally by experts for clients undertaking HCT. The form has four sections: The first section obtained personal information such as age, gender, marital status, number of children, number of wives, previous HCT and date of visit. The second section assessed clients' knowledge of HIV, the agent, mode of transmission and HIV prevention strategies. HIV risk assessment is the third section. This section obtained information regarding clients' sexual history within three months before HCT. History of casual unprotected sex, sex with a regular partner who is not a spouse, history of STI and sex with more than one partner. The last section obtained information relating to symptoms such as cough, weight loss, fever, night sweat, vaginal discharge and genital sores.

### Assessment of risky sexual behaviour

RSB is defined as a variety of behaviours which include; premarital sex, multiple sexual partners, unprotected sex, and others, which could result in contracting HIV/AIDS.^22^ However, in this study, RSB was defined as unprotected sex with a casual partner, unprotected sex with a regular partner who is not a spouse, STI and sex with more than one partner 3 months before HCT (as contained in the National HCT client intake form). An affirmative response to any of the four questions was considered as RSB. Clients were classified as not having RSB in the absence of all the four defining criteria.

### Data quality

Data were checked for errors, discrepancies, inconsistencies, completeness and missing information after data entry. The data captured were compared with the client's records to resolve inconsistencies and errors. Missing and incomplete data and other inconsistencies in the data set that could not be resolved were removed from the data set before data analysis.

### Data analysis

The study tool had a high content validity because it was reviewed nationally by experts on HIV, sexual and reproductive health. The Cronbach alpha coefficient for scales used to assess RSB was determined to be 0.81. Numerical variables were presented in percentages, mean and standard deviation. Outcome variables were HIV infection and RSB. Chi-square test was used to compare categorical variables while the Student ‘t’ test was used to compare continuous variables. Crude and adjusted odds ratios of factors associated with RSB were determined. The confidence interval was set at 95% and a p-value less than 0.05 was adjudged significant for all statistical tests. Statistical Package for Social Sciences (SPSS) IBM version 22 was used for data analysis.

### Ethical considerations

Approval for the study was obtained from the Health Research and Ethics Committee of the Lagos State University Teaching Hospital (Reg. No. NHREC 04/04/2008, approved on 31/07/2018).

## Results

A total of 4273 clients' records (95.4%) were analyzed out of 4478 clients that accessed the HCT services within the study period. About half (49.8%) were between 20 – 39 years, the mean age was 38.5±14.4 years (age range 5 – 85 years). There were more males (53.3%) than females (46.7%). The majority were married (59.2%), had previously done HCT (83.5%) and had previous sexual experience (90.9%) as shown in Table 1.

**Table 1:** Socio-demographic characteristics of clients accessing HCT

Variable	n = 4273	%
**Age group (years)**		
< 20	311	7.3
20 – 29	898	21.0
30 – 39	1229	28.8
40 – 49	915	21.4
50 – 59	501	11.7
≥ 60	419	9.8
Mean± SD	38.5±14.4	

**Gender**		
Male	2276	53.3
Female	1997	46.7

**Marital Status**		
Single	1416	33.1
Married	2531	59.2
Divorced	45	1.1
Separated	232	5.4
Widowed	49	1.1

**Previous HIV testing**		
Yes	3566	83.5
No	707	16.5

**Previous Sexual activity**		
Yes	3884	90.9
No	389	9.1

**Year**		
2016	1626	38.1

Table 2 shows the associated factors with unprotected sex with casual partner among study participants. The proportion of participants who had unprotected sex with a casual partners reduced with advancing age from 15.5% among clients aged less than 20 years to 1% among clients aged 60 years and above. Clients who had sex with a casual partner were younger (34.1±10.4) than clients who reported not having sex with a casual partner 3 months before HCT (40.6±13.6) p< 0.001. A higher proportion of males (6.4% vs 4.5%, p =0.009), HIV positive clients (8.5% vs 4.8%, p<0.001) and singles (12.1% vs 3.3% vs 1.8%, p<0.001) had unprotected sex with casual partner three months before HCT.

**Table 2:** Associated factors with unprotected sex with casual partner

Variable	Unprotected sex with casual partners		
	Yes (%)	No (%)	p
	n = 216	n = 3668	
**Age group (years)**			
< 20	11 (15.5)	60 (84.5)	
20 – 29	62 (7.5)	761 (92.5)	
30 – 39	93 (7.8)	1092 (92.2)	
40 – 49	31 (3.5)	865 (96.5)	
50 – 59	15 (3.0)	479 (97.0)	
≥ 60	4 (1.0)	411 (99.0)	
Mean±SD	34.1±10.4	40.6±13.6	<0.001

**Gender**			
Male	137 (6.4)	1992 (93.6)	0.009
Female	79 (4.5)	1676 (95.5)	

**Marital Status**			
Single#	133 (9.7)	1240 (90.3)	<0.001
Married	83 (3.3)	2428 (96.7)	

**HIV status**			
Positive	65 (8.5)	698 (91.5)	<0.001
Negative	151 (4.8)	2970 (95.2)	

**Previous HCT**			
Yes	177 (5.4)	3076 (94.6)	0.458
No	39 (6.2)	592 (93.8)	

NB: Clients who never had sex before HCT were removed before analysis

# comprised of singles, divorced, widowed and separated

HCT = HIV counselling and Testing

There was no age (p = 0.191) and gender difference (p = 0.201) in the proportion of clients that had unprotected sex with a regular partner other than a spouse. Marital status, HIV status and previous HCT were associated with unprotected sex with a regular sexual partner other than spouse three months before HCT (p<0.001) (Table 3). More clients with no previous HCT (6.5% vs 3.3%) had STI three months before HCT (p = 0.001) (Table 4). There was an association of age, marital status and previous HCT with sex with more than one sexual partner 3 months before HCT (p<0.05) (Table 5).

**Table 3:** Associated factors with unprotected sex with regular partner other than spouse

Variable	Unprotected sex with regular partner other than spouse		
	Yes (%)	No (%)	p
	n = 1352	n = 2532	
**Age group (years)**			
< 20	16 (22.5)	55 (77.5)	
20 – 29	223 (27.1)	600 (72.9)	
30 – 39	477 (40.3)	708 (59.7)	
40 – 49	378 (42.2)	518 (57.8)	
50 – 59	175 (35.4)	319 (64.6)	
≥ 60	83 (20.0)	332 (80.0)	
Mean±SD	39.8±11.4	40.4±14.7	0.191

**Gender**			
Male	760 (35.7)	1369 (64.3)	0.201
Female	592 (33.7)	1163 (66.3)	

**Marital Status**			
Single#	209 (15.2)	1164 (84.8)	<0.001
Married	1143 (45.5)	1368 (54.5)	

**HIV status**			
Positive	186 (24.4)	577 (75.6)	<0.001
Negative	1166 (37.4)	1955 (62.6)	

**Previous HCT**			
Yes	1157 (35.6)	2096 (64.4)	0.001
No	195 (30.6)	436 (69.1)	

NB: Clients who never had sex before HCT were removed before analysis

# comprised of singles, divorced, widowed and separated

HCT = HIV counselling and Testing

**Table 4:** Associated factors with STI three months before HCT

Variable	STI in the last 3 months		
	Yes (%) n = 147	No (%) n = 3737	p
**Age group (years)**			
<20	4 (5.6)	67 (94.4)	
20 – 29	34 (4.1)	789 (95.9)	
30 – 39	45 (3.8)	1140 (96.2)	
40 – 49	36 (4.0)	860 (96.0)	
50 – 59	18 (3.6)	476 (96.4)	
≥ 60	10 (2.4)	405 (97.6)	
Mean±SD	39.9±12.6	40.3±13.6	0.237

**Gender**			
Male	80 (3.8)	2049 (96.2)	0.922
Female	67 (3.8)	1688 (96.2)	

**Marital status**			
Single#	57 (4.1)	1316 (95.9)	0.376
Married	90 (3.6)	2421 (96.4)	

**HIV status**			
Positive	30 (3.9)	733 (96.1)	0.812
Negative	117 (3.7)	3004 (96.3)	

**Previous HCT**			
Yes	106 (3.3)	3147 (96.7)	0.001
No	41 (6.5)	590 (93.5)	

NB: Clients who never had sex before HCT were removed before analysis

# comprised of singles, divorced, widowed and separated

NB: HCT = HIV counselling and Testing

STI = Sexually transmitted in fection

**Table 5:** Associated factors with having more than one sexual partner

Variable	Having more than one sexual partner		
	Yes (%)	No (%)	p
	n = 89	n = 3795	
**Age group (years)**			
< 20	4 (5.6)	67 (94.4)	
20 – 29	23 (2.8)	800 (97.2)	
30 – 39	32 (2.7)	1153 (97.3)	
40 – 49	16 (1.8)	880 (98.2)	
50 – 59	10 (2.0)	484 (98.0)	
≥ 60	4 (1.0)	411 (99.0)	
Mean±SD	36.4±11.7	40.3±13.6	0.003

**Gender**			
Male	54 (2.5)	2075 (97.5)	0.261
Female	35 (2.0)	1720 (98.0)	

**Marital status**			
Single#	45 (3.3)	1328 (96.7)	0.002
Married	44 (1.8)	2467 (98.2)	

**HIV status**			
Positive	22 (2.9)	741 (97.1)	0.223
Negative	67 (2.1)	3054 (97.9)	

**Previous HCT**			
Yes	63 (1.9)	3190 (98.1)	0.001
No	26 (4.1)	605 (95.9)	

NB: Clients who never had sex before HCT were removed before analysis

# comprised of singles, divorced, widowed and separated

HCT = HIV counselling and Testing

The prevalence of RSB was 41.5. The chance of having RSB reduced with advancing age, from 5.6 fold chance more (AOR 5,6 95%CI 3.2 – 10.0, p = <0.001) among patients < 20 years, to 2.2 fold chance more (AOR 2.2 95%CI 1.7 – 3.0, p<0.001) among patients aged between 50 – 59 years compared to patients aged 60 years and above. There was no gender difference in the odds of having RSB (AOR 1.1, 95%CI 1.0 – 1.3, p = 0.061) while clients who were single (single, divorced, separated, widow) had a 3.8 fold chance more of having RSB than clients who were married (AOR 3.8, 95%CI 3.2 – 4.6, p<0.001). The chance of having RSB was 90% (AOR 1.9 95%CI 1.6 – 2.3, p<0.001) more among HIV positive clients then HIV negative clients. There was no difference between the odds of RSB among clients who had no previous HCT and clients who had undergone HCT previously (AOR 1.0 95%CI 0.8 – 1.2, p<0.998) (Table 6).

**Table 6:** Factors associated with risky sexual behavior

Variables	Sexual behavior			
	Risky	Non risky	COR (95%CI), p	AOR (95%CI), p
	n = 1610 (%)	n = 2274 (%)		
**Age group (years)**				
< 20	24 (33.8)	47 (66.2)	1.7 (1.0 – 3.0), 0.048	5.6 (3.2 – 10.0), <0.001
20 – 29	297 (36.1)	526 (63.9)	1.9 (1.4 – 2.5), <0.001	4.3 (3.2 – 5.8), <0.001
30 – 39	575 (48.5)	610 (51.5)	3.2 (2.4 – 4.1), <0.001	4.7 (3.6 – 6.2), <0.001
40 – 49	423 (47.2)	473 (52.8)	3.0 (2.3 – 3.9), <0.001	3.9 (3.0 – 5.1), <0.001
50 – 59	196 (39.7)	298 (60.3)	2.2 (1.7 – 3.0), <0.001	2.2 (1.7 – 3.0), <0.001
≥ 60	95 (22.9)	320 (77.1)	1	1

**Gender**				
Male	899 (42.2)	1230 (57.8)	1.1 (0.9 – 1.2), 0.281	1.1 (1.0 – 1.3), 0.061
Female	711 (40.5)	1044 (59.5)	1	1

**Marital Status**				
Single#	361 (26.3)	1012 (73.7)	1.8 (1.5 – 2.2), <0.001	3.8 (3.2 – 4.6), <0.001
Married	1249 (49.7)	1262 (50.3)	1	1

**HIV status**				
Positive	354 (46.4)	409 (53.6)	1.3 (1.1 – 1.5), 0.002	1.9 (1.6 – 2.3), <0.001
Negative	1256 (40.2)	1865 (59.8)	1	1

**Previous HCT**				
No	259 (41.0)	372 (59.0)	1.0 (0.8 – 1.2), 0.829	1.0 (0.8 – 1.2), 0.998
Yes	1351 (41.5)	1902 (58.5)	1	1

NB: COR = crude Odds ratio, AOR = Adjusted odds ratio, CI = Confidence interval: HCT = HIV counselling and Testing

# comprised of singles, divorced, widowed and separated

Clients that had no sex before HIV Counselling and Testing were removed before analysis

The Prevalence of risky sexual behavior was 41.5%.

## Discussion

This present study assessed the prevalence of RSB and its associated factors among clients who assessed HCT in a secondary referral hospital in Lagos Nigeria. The prevalence of RSB in our study was 41.5%. Our finding shows the prevalence and risk of RSB reduced with advancing age, was higher among singles and HIV positive clients. Gender and previous HCT were not associated with RSB.

The proportion of clients having RSB was 41.5% in this study. Our prevalence for RSB is comparable to what was obtained in studies from Ethiopia (42.8%), Ghana (41.5%) 23,24 and many other countries in sub-Saharan Africa.^25^ Lack of personalized HIV information, socioeconomic reasons, peer pressure and lack of parental control may be responsible for this high prevalence.^25^

The youths below 20 years had the highest prevalence of RSB in our study. A similar finding has been reported in studies from Nigeria and other sub-Saharan nations.^21^,^27^–^30^ Multiple sexual partners, non-marital sex, unprotected sex and poor condom use are commoner among young people.^6^,^20^ In many African settings, sexual issues are rarely discussed between parents and their wards. This may be due to ignorance or fear that knowledge of prevention of unwanted pregnancy may make the children sexually active.^31^

The chance of RSB was 3.8 fold more among clients who were single compared with clients that were married. Also, a higher proportion of the singles had unprotected sex and multiple sexual partners in our study. This may be related to age because a majority (99.3%) of the youths less than 20 years in our study were singles. High-risk sex has been reported among widows and divorced women (classified as single in this study) from Sub-Sahara Africa who were desperately in need of financial support.^32^,^33^

The role of gender in RSB has been reported in many studies from sub-Saharan Africa.^21^,^29^,^30^,^34^ In our study, there was no association between gender and RSB contrary to the findings from Nigeria, Tanzania, Ethiopia and Zambia which showed the risk of RSB to be higher among men^21^,^23^,^34^–^36^. The reason for our finding is unknown, our study population may be a possible explanation. Our study site was a referral center for infectious diseases in Lagos state and all referred patients were offered HCT. It is believed that societal norms, beliefs and culture which differentiated the gender roles of males and females have been attributed to the higher prevalence of RSB among men. Unlike females, masculinity is associated with power and authority which requires men to be risk-takers, to condones multiple sexual partners and have early sexual debut which consequently increases their vulnerability to contracting STIs and HIV.^37^,^38^

Although HCT is used as a preventive tool against HIV in sub-Saharan Africa, the behavioural changes following HCT are still controversial. Contrary to the findings from Tanzania and Kenya, our study showed that there was no association between previous HCT and RSB.^11^,^12^,^39^ Systematic reviews and meta-analysis from sub-Saharan countries opined that HCT did not affect the sexual behaviour of clients who were seronegative at initial HCT and clients who did not participate in HCT. However, HCT was shown to modify the sexual behaviour of HIV discordant couples.^13^,^14^

Heterosexual intercourse accounts for over 80% of HIV infection in Nigeria and consequently, protective sex is the single most efficient measure to reduce the sexual transmission of HIV. In our study, unprotected sexual intercourse with a casual partner 3 months before HCT was associated with HIV infection. This is similar to what was reported in a study from southern Nigeria where a high proportion of clients did not use condoms.20 The use of condoms in sub-Saharan Africa involves complex social, cultural and gender-power dynamics which put condom use predominantly under the control of men and limits the ability of women to introduce condoms into their relationship. 40,41 Male dominance in a sexual relationship is the norm in Africa and their approval is central to the use of condoms.^42^ Factors such as accessibility to condoms, insufficient or absent information, sexual norms promoting male dominance and virility, knowledge about HIV transmission, attitude towards sex and association of condom use with promiscuity as well as interfering with pleasure have been associated with condom use in sub-Saharan Africa.^43^

## Limitations

RSB was based on a self-report of clients that accessed HCT at the study location and as such may not represent the actual estimate of RSB of clients. The information given by clients regarding their sexual history 3 months before HCT may not be accurate due to recall bias. The sexual history is viewed as very personal in our setting and some clients may not give an accurate account of their sexual history for social reasons. Also, we could not evaluate other factors associated with RSB such as the age of sexual debut and use of substance since the study was a review of secondary data. Missing and incomplete data are common scenarios in a review of records. Although incomplete data were excluded during data analysis, it may affect the evaluation of our outcome variables.

## Conclusion

The prevalence of RSB was high in this study. Age, marital status and HIV status were associated factors of RSB. The current campaign to reduce unprotected sex and HIV infection must be sustained and the people especially the youths should have access to HCT centers. More youth-friendly health services should be created and access to contraceptives should be improved to mitigate the effects of RSB especially among the youths.
